# The role of personalised professional relations across care sectors in achieving high continuity of care

**DOI:** 10.1186/s12875-021-01418-8

**Published:** 2021-04-14

**Authors:** Johanna Forstner, Jasmin Bossert, Aline Weis, Nicola Litke, Cornelia Strassner, Joachim Szecsenyi, Michel Wensing

**Affiliations:** Department of General Practice and Health Services Research, University Hospital Heidelberg, Im Neuenheimer Feld 130.3, 69120 Heidelberg, Germany

**Keywords:** Continuity of care, Care coordination, General practice, Hospital, Hospital admission, Hospital discharge, Information flows, Cooperation

## Abstract

**Background:**

High continuity of care has a positive impact on health outcomes, but insight into the mechanisms underlying this impact is limited. Information continuity, on which our study focuses, is especially important when relational continuity is not given, which is often the case at hospital admission or hospital discharge. The aim of this study is to provide insight into the information flows between general practices and hospitals in Germany, and to identify factors associated with these flows of information.

**Methods:**

This is a qualitative interview study in a purposeful sample of staff from hospitals and general practices (general practitioners, care assistants in general practice, hospital management, hospital physicians, and nursing staff). Interviews were conducted via telephone or face-to-face using a self-developed semi-structured interview guide. Stepwise systematic content analysis was used to structure collected material into themes and sub-themes that related to the study aim. Data was analysed by two researchers in several cycles, alternating between inductive and deductive approaches.

**Results:**

A total of 49 interviews were conducted. Duration of the interviews varies between 21 and 78 min (mean duration 43 min). Across all groups, more than two thirds of participants were female (*n *= 34, 69%). The analysis highlighted six interdependent main themes regarding factors that affect information flows between hospitals and general practices: organisational, legal, financial, patient factors, individual characteristics, and emotional & social factors. The latter theme emerged as particularly rich and was therefore divided into four subthemes: appreciation and understanding of the respective other, (intrinsic) motivation, socialisation, and relationships. Organised meetings and events were mentioned as strategies to address emotional and social factors.

**Conclusions:**

Digitalisation can facilitate information flows between care providers. However, knowing each other and good personal relations remain important for effective collaboration. Cooperation between all stakeholders is needed to aim to achieve continuity of care.

**Trial registration:** DRKS00015183 on DRKS/ Universal Trial Number (UTN): U1111-1218–0992. Date of registration 23/08/2018.

## Background

High continuity of care has a positive impact on several outcomes, including patient satisfaction with healthcare [1], the utilisation of health services such as emergency departments [2, 3], quality of life [4], the total number of hospital admissions [5] as well as the number of hospital admissions for ambulatory care sensitive conditions [4], the number of hospital readmissions, and medication adverse events [6]. However, insight into the mechanisms underlying these effects is limited.

Continuity of care is described as health care that is coherent and connected [7]. Definitions of the concept in the literature differ and the distinction from related concepts can be difficult [8]. In their widely used definition, Haggerty et al. [7] define continuity of care by referring to three core dimensions: relational continuity, informational continuity, and management continuity. Informational continuity is defined as ‘the capacity of information to travel with the patient and within the health system’ ([5]:p.590f.). Relational continuity refers to a longitudinal and trusting relationship between a patient and one or more health care providers, which is considered to be a core element of primary care. Finally, management continuity means that health care delivered by several care providers follows a shared care protocol and that services are delivered complimentarily. The experience of continuity of care might be described differently by patients and health care providers [7].

Our study focuses on one aspect of information continuity across care sectors: the transfer of information that has been accumulated in general practice to hospital, usually in paper-based or digitally recorded formats [9]. In this context, the importance of information continuity is high, as patients receive treatment and care in both the outpatient and inpatient sectors, and especially when emergency hospital admissions occur [10, 11].

Collected information, patient records, telecommunication, referral systems, and feedback by other care providers are essential building blocks for achieving information continuity [5]. In Germany, ambulatory physicians are encouraged to provide the hospital with relevant information about the patients’ medical history, as far as they have access to this kind of information. Amongst others, this includes information on chronic diagnoses, medication, or recent diagnostics [12]. Hospitals are encouraged to consider information about the patient before admission [13]. At the point of discharge, hospitals should provide general practitioners (GP) or other treating ambulatory care physicians with information on procedures conducted, diagnostic results, medication at discharge, and recommended care. In Germany, hospitals are obligated to issue discharge letters, at least a preliminary version, to patients [12, 13]. At any time point, information should be passed timely in order to be considered in the planning of further care and thereby ensure patient safety [6]. Obviously, not only the availability of information but also the quality of the information impacts on patient outcomes [10].

The health care landscape in Germany is characterised by fragmentation, resulting from decentralised governance and a strong separation between hospital inpatient care and ambulatory medical care. In Germany, office-based ambulatory care is provided by primary care physicians and by (other) medical specialists, e.g. cardiologists and orthopaedics. There have been several reforms attempting to overcome the resulting challenges, such as offering strong primary care (‘Hausarztzentrierte Versorgung’ with the GP as gatekeeper), disease management programs, and integrated care programs for specific patients and population groups. Still, the combination of fragmentation of health care and pending use of information technology such as electronic health records impede information flows between care providers [14, 15].

A growing body of literature focusses on discharge letters that are missing information, or are sent out too late, and about GPs not being noticed about discharge. Thus, despite legal obligations and mutual consent about the relevance of information flows [6], communication and information transfer during care transitions remain a challenge, even in health systems that are less fragmented and more advanced in terms of digitalisation [6, 16–18].

The aim of this study therefore is to provide insight into factors influencing flows of information between general practices and hospitals.

## Methods

### Study design

This qualitative interview study explored information flows between hospitals and general practices from the perspective of care providers in the context of the VESPEERA project (Improving Patient care across sectors: An admission and discharge model in general practice and hospitals, *Versorgungskontinuitaet sichern: Patientenorientiertes Einweisungs- und Entlassmanagement in Hausarztpraxen und Krankenhaeusern*).

The VESPEERA project aims at improving admission and discharge management in hospitals and general practices in Germany [19]. The intervention program includes intervention components in general practices and hospitals before admission, during hospital stay as well as before and after discharge, such as an admission letter, a telephonic conversation before discharge, and telephone follow-up for patients at high risk for rehospitalisation. A total of 7 hospitals (with approx. 30 participating departments) and 72 general practices were involved in the project. The implementation of the complex intervention program was accompanied by an evaluation of processes and outcomes. The aim of the process evaluation was to gain insight into the intervention fidelity, attractiveness and acceptance of the program as well as factors influencing implementation [20].

### Sampling strategy

This purposeful sample comprises GPs and Care Assistants in General Practice (*Versorgungsassistentin in der Hausarztpraxis*, VERAH), hospital management, hospital physicians, and hospital nursing staff from general practices and hospitals participating in the VESPEEERA project, as well as staff from hospitals not participating in VESPEERA. Participants had to be able to provide insight into the implementation or execution of the VESPEERA intervention and/ or into admission and discharge management.

Initial contact and invitation for participation in an interview was sent out via post directly to all GPs and VERAHs from general practices participating in the VESPEERA study. Within the VESPEERA project, each hospital named a contact person for any project-related communication. These contact persons were invited for participation and asked to distribute the invitations to any eligible staff within their hospital or hospital department. For recruitment of staff from non-participating hospitals, a list of hospitals outside of the intervention region was created through an online search. A total of 59 invitations were sent out to hospital management of eligible hospitals. If several hospitals belonged to one clinic association, only one invitation was sent out. When interest in participation was expressed via a fax coupon, potential participants were contacted via phone for study information. Participants were included in this study when they met the following inclusion criteria: professional affiliation with one of the named sample-groups, age 18 years and older, adequate skills of reading and speaking German language, and the ability to give informed consent. All participants gave their written informed consent prior to the interview. Inclusion of interviews for the purpose of the research question of this study was ended when saturation of codes and contents in the analysis of the interview data was reached. Non-participation was not documented, as the total number of eligible persons reached is unknown.

### Data sources

Qualitative interviews were conducted using a self-developed semi-structured interview guide. As the interviews were conducted within the VESPEERA process evaluation, the interview guide was designed with regard to its research questions. Topics addressed in the interview guide were possible consequences of the intervention, the working mechanism to achieve these effects, attractiveness of the intervention program, as well as contextual factors that determined the implementation of the intervention. The interview guide was not modified during data collection.

### Data collection

The interviews were conducted from September 2018 through September 2019. Interviews were conducted by the experienced female researchers and doctoral candidates JF, NL and AW (all around 30 years of age). JF has a background in health services research and implementation science. NL is a speech and language therapist and is trained in interprofessional health care as well as health services research and implementation science. AW has a background in social sciences and medical process management. With the aim of reducing bias and to guarantee neutrality, as some of the participants and researchers were familiar with each other through the implementation process, we tried to have interviews conducted by researchers who had previously had little contact with the participants. Interviews were conducted either as telephone interviews or face-to-face interviews, according to the participants’ preference. Face-to-face interviews were conducted at the participants’ workspaces in a separated room with no other persons present. All interviews were audio recorded, handwritten notes were taken during the interview by the respective researcher. Interviews were transcribed using simplified transcription rules, thus transcribed verbatim without paying attention to dialect or informal language/ slang. Transcripts were not returned to participants for comments or correction, no repeat interviews were carried out.

Prior to the interview, all participants filled-in a paper-based questionnaire on sociodemographic information. The questionnaire included questions on the participants’ age, sex, profession, years worked in their profession, and structural characteristics of the organisation they work at.

### Data analysis

No predefined theory was used. Instead, stepwise systematic content analysis was used to structure collected material into themes and sub-themes that related to the study aim. First, a preliminary framework of themes was inductively developed based on a first glimpse of the data. Then, a first cycle of deductive line-by-line coding of the interviews was conducted by the first author of this study. A further researcher (JB, female, 31 years old at the time of data analysis) not involved in data collection, who has a background in health services research, implementation science, interprofessional health care and nursing, then selectively checked codings. Subsequently, the two researchers met and discussed codes and themes, which resulted in an inductive refinement of themes. In a second cycle of coding, data was deductively recoded in-depth and to the refined themes. The codes in each theme where then analysed by the first author by summarising the themes described within the different groups of the sample and between groups. The two authors then met to discuss the final coding of all interviews and major findings. MAXQDA software Version 18 was used for data coding and MS Excel for data analysis.

The COREQ guideline was used for reporting of this study [21].

### Ethics and data protection

Ethical approval was obtained by the Ethics Committee of the Medical Faculty Heidelberg (S-352/2018) for the process evaluation of the VESPEERA study. All participants gave their written informed consent prior to study participation. Data were pseudonymised before data analysis.

## Results

### Description of the study population

A total of 49 interviews was conducted. Duration of the interviews varies between 21 and 78 min (mean duration 43 min). The assignment to groups can be found in Table 1. The groups are not mutually exclusive, the assignment to the groups therefore was based on the individual’s role in VESPEERA (not applicable for staff from hospitals not participating in the VESPEERA project). For instance, nursing staff who were appointed implementation leaders for the VESPEERA implementation were assigned to the hospital management group.

**Table 1:** Sociodemographic characteristics of participants in the qualitative interview study

	General practices		Hospitals			Total
	GPs	VERAHs^a^	Management	Physicians	Nursing staff	
Age	58 (50–64)* *n *= 6	40.5 (31–54)* *n *= 11	50 (29–60)* *n *= 15	53.5 (34–67)* *n *= 6	41 (21–61)* *n *= 11	47 (21–67)* *n *= 49
Sex (male)	2 (33%)** *n *= 6	0 (0%)** *n *= 11	7 (47%)** *n *= 15	5 (83%)** *n *= 6	1 (9%)** *n *= 11	15 (31%)** *n *= 49
Urban area	3 (50%)** *n *= 6	6 (60%)** *n *= 10	10 (66%)** *n *= 15	2 (33%)** *n *= 6	7 (64%)** *n *= 11	28 (60%)** *n *= 49
Years of experience	16.5 (2–25)* *n *= 6	17.5 (3–38)* *n *= 10	12 (2–22) *n *= 15	18 (7–32) *n *= 6	11 (0.2528) *n *= 11	14 (0.25–38)* *n *= 48
Single practice	4 (67%)** *n *= 6	5 (50%)** *n *= 10				9 (56%)** *n *= 16
Practice size (patients per quarter year)	1467 (850–2400)* *n *= 6	1775 (999–3000)* *n *= 8				1643 (850–3000)* *n *= 14
Hospital size: basic and regular care^b^			6 (40%)** *n *= 15	4 (67%)** *n *= 6	7 (64%)** *n *= 11	17 (53%)** *n *= 32

^*^mean (min–max), ** Frequencies (percent)^a^ care assistant in general practice^b^ In Baden-Wuerttemberg (our setting), hospital supply used to be based on the division of hospitals into categories, based on the number of beds. There is a distinction between basic, regular, specialized, and maximum care (e.g. provided by university hospitals). Basic care hospitals only provide some highly used inpatient services, such as obstetrics, emergency care, and internal medicine. Albeit no longer used, this categorisation still gives an impression of the size and geographical reach of a hospital [22]. The number provided refers to hospitals that belong to the categories basic and regular care

Across all groups, more than two thirds of participants were female (*n *= 34, 69%). The median age was 47 years, ranging from 21 to 67. Based on the district of their organisation, about two thirds of the participants worked in an urban area (*n *= 28, 60%). Table 1 provides an overview of further sociodemographic characteristics of the study population.

### Results of the qualitative interview analysis

First, an overview on current and desired information flows as reported by the interview participants will be given. Then, an overview on the themes identified in the analysis will be presented. Finally, an outlook on strategies to address some of the topics will be given.

#### Current and optimal information flows

The interviews showed that information between hospitals and general practices is mostly transferred via written means (such as doctors’ letters, medication plans or laboratory results at admission, or discharge letters) with the patient being the one to hand over the documents to the other care provider. For direct transfer between health care organisations, fax is used in most cases. Oral communication via telephone does happen in some cases, for example before an acute hospital admission, but rarely around discharge. The frequency of contact and the quality of documents passed is very heterogeneous. Participants see a need for improvement of information flows.

Regarding information flows in the future, participants wish for a standard that defines the means of communication for different situations. Ideally, health care organisations can communicate with media continuity where admission letters and discharge letters are transferred in the same and standardised way. Both sides wish for notifications about hospital admissions and discharges. A messenger system could be used for such notifications that do not require an answer or reaction. Furthermore, GPs and hospital staff had several ideas about how communication should be digitalised. They mentioned software that should be compatible for information exchange, shared platform such as electronic health records or referral platforms, or allowing GPs access to hospitals’ information systems. Only few participants did not see a need to change or would rather stick with paper-based communication. All participants said that they do receive phone calls, however not often enough:

"I don't necessarily see that there are more phone calls now either…no, rather less. I'm rather disappointed, to be honest. […] So it hasn't gotten worse, but it hasn't gotten better either." (general practitioner).

The analysis highlighted six interdependent main themes regarding factors that affect information flows between hospitals and general practices: organisational, legal, financial, patient factors, individual characteristics, and emotional & social factors. The latter was further divided into four subthemes. Table 2 provides an overview on all themes identified.

**Table 2:** Categories of factors associated with information flows

	**Definition**	**Examples of topics identified**
Organisational	Statements on the extent to which organisational (outpatient, inpatient, cross-sector) factors influence information flows	-Information systems in hospitals and general practices are not compatible -Phone calls mostly from smaller hospitals -Different timelines in hospitals and general practices impede telephonic reachability (a.m. surgery times in hospitals, p.m. out-of-office hours in general practices) -Telephone calls disrupt current activities -In general practices that are well organised, medical assistants work independently and manage information flows -Skills mix improves communication: case management, medical assistants, physician assistants
Legal	Statements about the extent to which legal requirements influence information flows	-Data protection (fax requires phone calls for verification of identity, no verification of identity possible via telephone, impedes digital communication, hospitals need patients’ consent for transferring medical information to other care providers) -Statutory regulations do not apply to patients with private health insurance (e.g. GP does not receive a discharge letter)
Financial	Statements on the extent to which financial factors influence information flows	-Health care organisations have to implement communication and information technology at their own financial risk -Communication is not financially compensated
Patient factors	Statements about the influence of patient factors on information flows	-Communication is more intense in complex cases (e.g. patients with multimorbidity, polypharmacy, geriatric patients, oncological patients) or if misunderstandings are expected to occur -Communication via telephone is preferred in those cases
Individual characteristics	Statements about the extent to which individual characteristics influence information flows	-Some physicians are more likely to call than physicians in other hospitals -Language barriers with foreign staff hinder information exchange
Emotional & social factors		
Appreciation and understanding of the respective other	Statements about how understanding of the tasks of others and appreciation for their work influences information flows	-GPs do not feel appreciated by junior physicians in hospitals, GPs are under the impression that they put all the work on the GP -VERAHs feel that detailed information at admission is not appreciated by the care providers in hospitals -Hospital physicians tend to not trusting external diagnostic results -Communication improves when care providers have worked together for a longer time, as they gain understanding about the others’ working mechanism -Work shadowing can be an opportunity for developing an understanding for different areas in health care
(Intrinsic) motivation	Statements about what influence (intrinsic) motivation has on information flows	-Only few individuals seem to be intrinsically motivated to communicate with other care providers -Motivation could be improved through positive reinforcement by patients -A change in paradigm is needed for hospital staff: health care does not end with hospital discharge
Socialisation	Statements about how information flows are influenced by different types of socialisation	-Medical students outside of Germany seem to be better trained for information exchange with other health care providers -Participants experience information flows to be better among geriatricians or oncologists -Young GPs who have spent time in hospitals seem to be more aware about the information that needs to be passed -Differences are observed between physicians in big hospitals, small hospitals, or general practices
Relationships	Statements on the extent to which personal relationships and contacts influence information flows and contact establishment	-Knowing someone and having met someone positively influences information flows and contact establishment -Knowing someone especially influences the probability of oral communication -Care providers know each other from formal and informal meetings, both are used for professional exchange -Knowing someone seems to be one of the or the most important factor for successful information flows

#### Organisational factors

This main theme represents several organisational aspects that participants think affect information flows between hospitals and general practices and that mostly refer to oral communication via telephone.

One that has been commented on by participants from all groups except GPs is reachability of the respective other, amongst others due to the fact that GPs and hospitals have different timelines:

‘The different time lines where you are in the hospital and in the practice […] when rounds take place and the case managers become active, that…that just doesn't fit to the processes in the general practice.’ (hospital management).

Hospital management and VERAHs had the impression that telephone calls are impacted by time constraints, for example due to lacking personnel.

Hospital physicians saw telephone calls as a problem as they cause them to interrupt their ongoing activity. A solution to this problem, according to hospital management, is the implementation of contact persons, designated time slots for telephone calls between different care providers, or simply sending mails with a request for a call back.

Furthermore, VERAHs were under the impression that whether hospital staff calls the general practice depends on the organisation. According to their experience, staff from smaller hospitals rather call than staff from bigger hospitals such as university hospitals:

‘Well, I don't think that in the big hospitals the physicians are going to start calling [us]. I don't think so. I think it's more likely to happen in smaller hospitals.’ (VERAH)

Another factor impacting information flows between hospitals and general practices is the skills mix, as described by hospital management and nursing staff. Case management staff, medical assistants on hospital wards or physician assistants can help to improve information flows with general practices:

‘If it’s about something that’s specifically medical or rather nursing related, they take over those calls and that way communication is better, yes.’ (nursing staff).

Considering general practices, hospital management found well organised practices to be characterised by competent medical assistants.

Lastly, GPs commented on the fact that most health care providers use different information systems, which complicates information flows.

All in all, participants from all groups made comments on how organisational aspects impact information flows from and to other health care providers. Hospital management staff were the only ones who took a critical view on their own organisation and an appreciative view on other organisations. Participants from the other groups rather suggested difficulties within organisations from other health care sectors.

#### Legal factors

The majority of comments in this subtheme addresses data protection as a factor that affects information flows between health care providers. Care providers often communicate via telephone or fax. Both are impacted by data protection measures, as described by all participants except GPs and hospital physicians. Faxing was seen critical from a data protective perspective, as the sender does not know whether the documents are received by the correct recipient. Therefore, information passed via fax is usually accompanied by telephone calls for the purpose of identity verification:

‘And then you have to call and then a fax is sent here and you have to confirm again that it really is you, and back and… Data protection, no, you know everything. Well, it’s always an act.‘ (VERAH)

However, even on the telephone the counterpart is unknown, as was mentioned by hospital management. Nursing staff mentioned that they need the consent by patients to exchange information about the patient with other care providers. GPs in contrast do not receive a discharge letter if only the patient is listed as recipient. Hospital management and physicians also found that data protection impedes digital information flows, for example via a common platform.

Other legal factors include a patients’ health insurance: one GP said that for patients with compulsory health insurance, hospitals are obligated to write a discharge letter. These obligations do not apply for patients with private health insurance and here, he often does not receive any information at all.

#### Financial factors

Only two participants from hospital management mentioned financial factors that impact information flows between care providers.

One participant said that as long as there are no specifications for a telematics infrastructure by the government, health care organisations have to implement structures at their own financial risk:

‘With the risk that we will have to spend money on this and switch over at some point as the IT infrastructure outside the hospital evolves’ (hospital management)

Another participant went one step further and said that not a minute that care providers invest into information flows is compensated.

#### Patient factors

The discharge letter is the preferred means of communication for patients with a ‘normal’ course of treatment. Participants from all groups agreed that information exchange is more intense and rather like collaboration, and therefore more often happens via telephone, for complex cases such as multimorbidity, polypharmacy, complex medication, palliative patients, patients with wound healing disorders, patients with parenteral feeding or an analgesic pump, geriatric patients, oncological patients, patients who reside in long-term care institutions, cases that require coordination of care after discharge, or patients who have had a long hospital stay.

‘From time to time, it happens that patients whose discharge situation is difficult or complex and we know that some problems are still shaky, well, are uncertain how it will develop further, we try to make telephone contact with the doctors who will continue to care for them beforehand, so that we say "This and that were our considerations. We assume that it will continue like this, but there could also be problems". That the attending general practitioner simply is also informed by telephone, and can perhaps also consider whether he will then carry out the follow-up care more closely and see the patient the next day, if there are home visits.’ (hospital physician).

Hospital physicians added that other cases where they would call a GP before discharge include those where misunderstandings are likely to occur. Another example would be patients who have comprehension problems regarding their own condition.

Only few participants from the group of VERAHs said that they do not see any patient characteristics to be influencing information flows with hospitals.

#### Individual characteristics

Participants from hospital management and hospital physicians mentioned that the degree of information flow depends on the physician himself. One hospital manager made this observation for GPs and mentioned that some even visit their patients in the hospital. One hospital physician spoke for his own group and said that there are some physicians who would always call the GP whereas other physicians would never call:

‘So I could tell you that there are colleagues who always call because they think it makes sense, because they want to pass on their needs or specific questions to the doctor who is responsible for the following care. Yes, and there are colleagues who never do that, so to speak.’ (hospital physician).

One VERAH added that with foreign physicians, language barriers often hinder communication.

The participants did not give any further detail on which other individual characteristics affect information flows.

#### Emotional & social factors—Appreciation and understanding of the respective other

Participants from all groups except hospital nursing staff commented on the issue of appreciation and understanding of the respective other and how this influences information flows.

GPs reported that that they do not feel appreciated by hospital staff. They were under the impression that all work is put to them by hospital staff. One GP especially did not feel appreciated by junior physicians:

‘How they (the junior physicians) judge us general practitioners. – ‘Look at this and how and what he's sending us today’ or something < both laugh > ‘just gives us work’ < laughs>.’ (general practitioner).

Furthermore, GPs wished to receive more information about their patients and would like to be up to date regarding their patients’ health.

VERAHs made the experience that providing paramedics and hospitals with admission letters is not appreciated. They were unsure whether admission letters even find consideration in the hospital.

One participant from hospital management said that hospital physicians do not trust external diagnostic results. Another participant said that after having cooperated with other care providers for a while one knows the working methods and requirements of the other. This improves information flows.

Hospital physicians see the attitude among their colleagues that in many cases it is appropriate to involve GPs as they do know their patients well. One participant sees opportunities in including work shadowings for medical students to develop an understanding for different areas in health care*.*

All in all, each sector was critical about the respective other sector.

#### Emotional & social factors—(intrinsic) Motivation

Two participants from hospital management commented on motivation being a factor to affect information flows. One said that it is only individuals who are motivated. Another participant saw the need for a change of paradigm. He said that among hospital staff awareness must be raised about the fact that health care does not end with a hospital discharge. He sees an opportunity to increase motivation through positive reinforcement.

‘Well, I just think that the cultural change in people's minds, […] the fact that people are moving towards each other, is very important.’ (hospital management)

#### Emotional & social factors—Socialisation

One GP was under the impression that universities outside of Germany prepare medical students better for information exchange with other health care providers. The same participant said that he attaches the quality of communication to the medical discipline. He observed good cooperation among geriatricians and oncologists.

VERAHs described that young GPs who used to work in a hospital, for example during their specialist medical training are more aware about which information is relevant in the hospital.

‘We have two young doctors who have been in the clinic for a long time, who come from a clinic, and they are totally aware of the fact, they say you need the reports.’ (VERAH)

Hospital physicians also reported about the relevance of knowing the other care providers’ or organisations’ workflows and see the need to spend time in different organisations in during medical training. Furthermore, they said that medicine is practiced differently in bigger hospitals than in smaller hospitals or general practices.

Hospital management staff mentioned that they have made the experience that achieving several certificates improved the cooperation with their admitting physicians. They also said that one has to consider that different health care providers such as GPs or outpatient specialist physicians need to be approached differently.

There were not many statements, but there were complementary comments from participants from all groups.

#### Emotional & social factors—Relationships

There were many statements, mostly by GPs, hospital management and hospital physicians, on the positive influence of knowing someone personally on information flows and especially oral communication via telephone. One hospital physician sees anonymity as a barrier to contact other care providers. Others said how knowing someone, or better knowing someone’s face, makes it easier to pick up the phone and call them:

‘You could clearly tell that when you are sitting together at one table and have seen each other, you pick up the phone faster, get in touch personally and some things simply run more smoothly’ (hospital management).

Participants talked about where they know other care providers from. GPs and VERAHs mentioned quality circles as an opportunity to get to know other health professionals. Other opportunities are events organised within physicians’ networks (such as Christmas parties), groups of regulars or round tables. These events are seen as possibilities to get to know each other, meet new people, intensify relationships with established contacts, or even make friends. Furthermore, private and informal meetings are used for professional exchange.

‘Well, it was initiated by our former managing director here from the [health insurer] in [city] and he is retired but still on the road as a consultant, I know him well from back in the day and we had dinner together <laughs >.’ (hospital physician).

GPs said that they are more connected with senior physicians than with assistant doctors, the latter probably do not see the relevance, according to GPs. Furthermore, they do see a need to get more connected among GPs. On the contrary, one participant from hospital management said that he has friends in other hospitals and he does make use of those contacts for information exchange.

Hospital physicians and GPs agreed that they would rather call someone if they know them. Nursing staff added that this also applies for social services. The probability of oral communication somewhat depends on the individual or rather the relationship between individuals:

‘You know what you look like, you know each other, you sometimes talk about something else [than work], you sit together over a beer and in this respect there's a good contact […] Every now and then he calls and says, for example, ‘I've got this and that, you sent me this and that and I did that’, that was—like that and like that and that's all right or you have to look again or something, it's going quite well, yes.’ (general practitioner).

One hospital physician as well as a nurse added that this is especially the case in smaller cities, where people know each other:

‘Got a face to go with it, right. [City] is not exactly a big city, which means you know each other […] and we know how to take each other, that's certainly an advantage.’ (hospital physician)

One participant said that information flows mostly depends on knowing others, therefore any other efforts on improving information flows are not worthwhile. Others supported this by suggesting strategies for improving cooperation such as intensifying established relationships or implementing permanent contact persons who always take calls.

#### Strategies to address emotional and social factors

The participants mentioned organised meetings and events as strategies in order to address some of the emotional and social factors.

Hospital management staff and hospital physicians commented on organised meetings and events as an opportunity to exchange ideas with other health care providers:

‘I mean, here at the university hospital, it's the same as in [place]. You could attend any kind of continuing education events from early morning to late at night, which are of course also open to ambulatory physicians, but they are often very specific to complex diseases. In [place], as well as in [place] I, there is the general practitioners' day once in half a year, where also interested people can come. So such events, I think, could help to lower or break down obstacles and barriers.’ (hospital physician).

These could serve as platforms to introduce themselves to others, to exchange views on positive and negative aspects about the cooperation between the organisations involved, and to discuss the stakeholders’ roles and responsibilities. One hospital physician said that networking among hospitals is also important.

The participants mentioned different events that could be adequate platforms for the described exchange: quality circles, continuing education events (such as ‘Day of General Practice’, which is established with departments of general practice in university hospitals in Germany and should be open for hospital physicians as well), or yearly meetings with other care providers. The latter is a kind of event that hospital management described to be established with nursing staff in hospitals together with other long-term care institutions.

## Discussion

This study originally focused on information continuity between primary care and hospitals, but also enabled a digression to management continuity. Information flows seem to be influenced by many structural factors such as organisational and data protection issues. In addition, social factors were prominent among the factors that influence information flows. Even in times of increasing use of modern information technology, factors such as appreciation of the other’s responsibilities and personally knowing each other were perceived to be crucially important for information flows and effective collaboration.

Organisational issues affect information flows, such as different work rhythms in hospitals and general practices that affect reachability via telephone. Similar results were reported by Jones et al. [23]. One may need several attempts in order to reach the desired person, which leads to time constraints in an inherently tight work schedule. Time constraints as a barrier to communication and cooperation were also mentioned by Bramesfeld et al. [24], which goes hand in hand with financial aspects. The participants in our study proposed to introduce timeslots that are intended for telephonic conversations between general practices and hospitals to overcome this problem.

Data protection hinders information flows between care providers as it leads to uncertainty about which means of communication to use. Even though fax machines lost their importance with the rise of e-mail in the twenty-first century, fax still seems to be the preferred means of communication among health care professionals in Germany [25, 26]. By the participants, it is seen as an aspect of convenience. However, in most cases faxes are accompanied by a phone call to ensure data protection, which again leads to time constraints.

Only few participants of this study state that they use e-mail or shared platforms as a means of communication. This reflects Germany’s position as a relative laggard in digitalisation in health care. Information and communication technology is in the early stages of a national roll-out with a delay in protocol of approximately ten years compared to some other countries [27]. Therefore, care providers resort to other means of communications and accept the risks associated. The current status of digitalisation thus leaves room for adaptions of the interventions. The participants mentioned inter and intra organisational messaging services for messages that do not need a synchronous response. This should be considered by health policy makers. Other studies also consider messaging services to be a suitable tool [28].

Technological infrastructures and organisational changes can facilitate information flows between care providers, but these were not the only important preconditions according to the interviewed individuals. Participants described emotional and social aspects as highly affecting information flows. First, this includes an understanding and appreciation of the respective others role and responsibilities, as well as an idea about what the other needs in terms of information at a certain time point. This is in accordance with previous research [29]. Second, participants from all groups stress the importance of knowing their counterpart as one, if not the most important (as mentioned by one participant), factor to successful information flows. Different stages of knowing someone lead to different amounts of effort put into information flows: if I know someone’s face, I am more likely to provide them with relevant information. If, however, I have a personal relationship with someone, I am more likely to not only communicate about a patient’s case, but to collaborate with them and to involve their opinions and expertise when planning patient care. Similar results were found by Bramesfeld et al. [24] for cooperation in mental health care, as well as by Jones et al. [23] and Munchhof et al. [28] for communication between general practitioners and hospital physicians. Results by Scaioli et al. [30] and Vargas et al. [31] confirm knowing someone personally as a factor that influences the provision of referral letters in between general practitioners and outpatient specialists are similar. Both studies were conducted in large cross-sectional studies in Latin America and Europe, respectively.

In our study, this mostly applied to synchronous communication via telephone. However, even in countries where information and communication technology is more advanced, deficits in information transfer during care transitions persist [32]. Furthermore and complementary, Munchhof et al. [28] found that direct communication, via phone, mail or a message, can help to pay more attention to discharge letters.

Price and Lau [33] link the relationship between care providers, which they call provider connectedness, to the concept of continuity of care. They found that communication is more effective and continuity of care is more likely to be achieved when care providers built on an existing relationship. The authors describe communication as the ‘glue’ ([33]:p.310) that facilitates continuity of care. Provider connectedness can emerge when care providers are geographically close or if they share patients over long periods of time. The authors also mention how some care providers take action in order to build good relationships. Our results suggest that ‘knowing each other’ seems to be inherent to rural areas, but it may also be achieved in more urban areas where health professionals do not naturally know each other. If this is the case, health care organisations are recommended to endeavour to create these relationships.

Nevertheless, considering high rates of fluctuation in hospitals, especially among physicians, relationships should not only be built between individuals in organisations. Instead, cooperation should happen on an organisational level and should include agreements on the form of communication in different situations, such as asynchronous and standardised communication in routine cases or synchronous communication (face-to-face communication, via telephone) in complex cases that deviate from routine. Moreover, cooperation should include clarification about the roles and responsibilities of different care providers in the process of patient care. The participants suggested to organise meetings and events between GPs and hospitals in order to connect care provider. They could also help with gaining understanding and growing appreciation for the respective other and their responsibilities. Medical education, where physicians in training see different areas of health care, and work shadowings can be complementary strategies.

The results of this study highlight the relevance of personalised professional relations for the interplay of the different dimensions of continuity of care. Relational continuity with a longitudinal and trusting relationship should antecedent to informational continuity, as it helps with accumulating information about a patient. However, this involves patients and care providers, which was out of focus of our study. The accumulated information is then shared between providers and necessary in order to provide care complementarily. The interview partners implied this by mentioning that information about the patient’s situation before hospital admission can help with planning care after discharge to fit the patients’ context. Personal relations between care providers facilitate those information flows, especially those that can be considered direct and synchronous, such as telephone calls. This is not only desired by care providers, but also the foundation for collaboration and thus management continuity.

There are some limitations to our study. First, there might be a selection bias. The sample of participants was mostly recruited from organisations who participate in the VESPEERA project and therefore might have a higher readiness for change regarding admission and discharge management. Furthermore, the interview guide was not primarily designed with regard to the research question of this study, but the data emerging from the interviews appeared to facilitate the study.

Our study adds to a large body of research on care transitions [34]. Only few of those papers have highlighted the relevance of personal relations to information transfers between health care organisations. The strength of our study is that we link it to continuity of care, especially information and management continuity, a dimension of continuity of care which is often neglected in continuity of care studies [35]. The results of this study stress how all dimensions need to be addressed in order to increase continuity of care.

## Conclusion

Digitalisation can facilitate information flows between care providers, but knowing each other and good personal relations are at least as important for good information flows and effective collaboration. Cooperation between all organisations who are involved in patient care is needed in the form of an alignment of processes and responsibilities. Only then, continuity of care in its several dimensions can be aimed to achieve.

## Data Availability

Data are available upon request with the authors.

## Abbreviations

GP: General practitioner
VERAH: Care Assistants in General Practice (*Versorgungsassistentin in der Hausarztpraxis*, VERAH
